# Supplementary material for: Content-rich biological network constructed by mining PubMed abstracts
Source: BMC Bioinformatics. 2004 Oct 8;5:147. doi: 10.1186/1471-2105-5-147 (PMC528731; doi:10.1186/1471-2105-5-147)
Supplement: Additional File 2 — The original results of the above study (non-essential files are deleted to keep the file size under the limit set by BMC bioinformatics). [file 1471-2105-5-147-S2.bz2 › chilibotAdditionalFile2/dip05/105ID8383120E439/html/MYOD1_TCF3.html]

 


 **MYOD1** and **TCF3** 
  
Found 43 abstracts in PubMed, retrieved 05.  
 

 What does Google say? 
 PDF only 
| .edu only 

---

**Interactive relationship** (e.g. stimulation, inhibition, etc)

**Neutral relationship**- Furthermore, we found that MyoD  [ **MYOD1** ]  co transfected with either ITF  [ **TCF3** ]  2B or ITF  [ **TCF3** ]  2A converted fibroblasts into myoblasts with the same frequency.  Ref: 10833525 J Biol Chem, 2000
- MyoD  [ **MYOD1** ]  heterodimerizes with E type factors E12 E47 and ITF  [ **TCF3** ]  2A ITF  [ **TCF3** ]  2B and binds E box sequences within promoters of muscle specific genes.  Ref: 10833525 J Biol Chem, 2000
- In transient transfection assays, MyoD  [ **MYOD1** ]  activates transcription in the presence of ITF  [ **TCF3** ]  2A but not ITF  [ **TCF3** ]  2B, which contains a 182 amino acid N terminal extension.  Ref: 10833525 J Biol Chem, 2000

**Non-interactive relationship** (e.g. studied together, co-existance, homology, etc.)

- In these cells, which displayed unchanged levels of the ubiquitous basic helix loop helix E2A  [ **TCF3** ]  factors and Id proteins, Tax was found to target the muscle specific basic helix loop helix transcription factor MyoD  [ **MYOD1** ] .  Ref: 11751456 Cell Growth Differ, 2001
- Analysis of the inhibition of MyoD  [ **MYOD1** ]  activity by ITF  [ **TCF3** ]  2B and full length E12 E47.  Ref: 10833525 J Biol Chem, 2000
